# The social life of self‐injury: exploring the communicative dimension of a very personal practice

**DOI:** 10.1111/1467-9566.12994

**Published:** 2019-09-25

**Authors:** Peter Steggals, Steph Lawler, Ruth Graham

**Affiliations:** ^1^ School of Geography, Politics and Sociology (Sociology) Newcastle University Newcastle upon Tyne UK; ^2^ Department of Sociology University of York Wentworth College York UK

**Keywords:** nonsuicidal self‐injury, self‐harm, social interaction, sociology

## Abstract

This article makes the case for a sociological focus on the communicative, relational and interactional dimensions of nonsuicidal self‐injury. While current research tends to be dominated by highly individual and intrapsychic models, it is increasingly observed that such models leave a social dimension to the practice unexplained. A burgeoning sociological literature has begun to address this paradox of the social in self‐injury; however, we argue that the role of the social must be considered beyond the issues of aetiology, social learning and social construal/construction that are typically covered in this literature. Specifically, we argue that, since the lived meanings of self‐injury directly implicate the interactional along with the intrapsychic, a more systematic focus on the role of social relations and social communication is vital. To illustrate this conceptual argument and embed it in the lived experiences of self‐injury, we draw on two case studies taken from pilot research conducted by the authors. The more thoroughly sociological approach to self‐injury that we present here offers an important compliment to the existing evidence base by reframing the absent presence of social communication contained within it, and suggesting important future directions for research.

## Introduction

Nonsuicidal self‐injury has long been thought of as a peculiarly enigmatic practice (Muehlenkamp *et al*. 2012), something ‘confused and confusing’ to use Pierce's memorably resigned phrasing (1977: 377). A key aspect of this ambiguity centres on the way that social and communicative elements persistently haunt what is otherwise considered an intensely private matter (Chandler 2016, Chandler *et al*. 2011, McShane 2012, Steggals 2015). Descriptions of self‐injury as a ‘form of violent *communication*’ (Grocutt 2009: 105), a ‘system of *signs* marking *statements* about the self’ (Gardner 2001: 4) and a ‘*language* of blood and pain’ (Hewitt 1997: 58) voicing ‘things that cannot be [otherwise] said’ (Pembroke 1996: 45) are quite common (our emphasis throughout). But these descriptions exist in a pronounced and unresolved tension with the dominant models of self‐injury that typically frame it as a wholly and claustrophobically personal crisis; something completely ‘inner’ and therefore *not* ‘outer’, something individual and therefore *not* social and something private and therefore *not* interactional (Chandler 2016, Chandler *et al*. 2011, Steggals 2015). We call this tension the paradox of the social in self‐injury.

Recently, the dominance of this wholly intrapsychic and individualistic framing of self‐injury has been challenged by sociological and historical work that has produced important insights into its social aetiology, social learning and social construal/construction (Adler and Adler 2007, 2008, 2011, 2012, Brossard 2014, Chandler 2012, 2013, 2014, 2016, Chandler *et al*. 2011, 2016, Chaney 2017, Frost 2001, Hodgson 2004, Inckle 2007, 2014, Kilby 2001, McShane 2012, Millard 2015, Steggals 2015). However, while this work has helped to fill in the social background to self‐injury, nonetheless, much like the dominant models it critically engages with, it still tends to foreground the figure of the psychosocially isolated and uncommunicative individual. Meanwhile, the paradox of the social in self‐injury suggests directly *social communicative, interactional* and even *relational* dimensions to self‐injury. As such, we argue that the implication of the paradox is that our understanding of self‐injury will always be necessarily partial as long as we restrict ourselves to this figure of the uncommunicative individual. In order to develop a fuller understanding of self‐injury, it is vital that we explore its social communicative dimensions.

The relative neglect of this aspect of self‐injury (Muehlenkamp *et al*. 2012: 67–8) may in part be due to a lack of sociological imagination in the psy‐centric paradigms that dominate research. But another factor that has strongly militated against this kind of research (including sociological work) is a common concern that suggesting self‐injury is in any way relational is tantamount to dismissing it as ‘*attention‐seeking’*; a pejorative phrase implying an illegitimate and histrionic social manipulation (Chandler 2016, Pembroke 1996). As we discuss later, self‐injury is frequently assumed to only be *authentic* when it is *non‐communicative*. Part of our task then, is to think about how self‐injury can be both an intensely personal practice *and* something that is suffused with social processes; an authentic expression of genuine distress, while at the same time something intertwined with, and not separate from, the interaction order (Goffman 1983). As such, we argue in this article that self‐injury requires, in the words of Goffman's deeply relevant study of stigma, a ‘language of relationships, not attributes’ (1968: 13).

To make our case, we begin by surveying the status of the social in existing theory and research and establish the social communicative as a kind of absent presence within this work. In our second section, we examine the issue of ‘attention‐seeking’ as the primary obstacle to further research on social communication in self‐injury, and we explore the issues of *visibility* (Chandler 2016: 109–44) and *recognition* (Frank 1991: 87, Fraser and Honneth 2003) which, while often underdeveloped in self‐injury research, represent promising ways into a more interactional and relational approach.

While ours is an exploratory article, intended to review the existing evidence base and develop from it suggestions for new directions in research, the lived reality of self‐injury is always personal, situated and intimately tied to narrative and experience (McShane 2012). As such, sections three and four draw on two complementary case studies to illustrate our argument and contextualise it within the details of actual lived experience. These case studies are drawn from a 2016–2017 English pilot study carried out by the authors. The study explored the degree to which significant relationships shape people's practices and experiences of self‐injury, even as these relationships are themselves affected and shaped by self‐injury. Following Klonsky and Muehlenkamp's claim that an individual might self‐injure to elicit responses from a significant other and yet not be ‘fully aware that their self‐injury is encouraged or reinforced by its effects on [these] others’ (2007: 1050), our pilot study gathered data from 26 in‐depth semi‐structured qualitative interviews (20 with female participants, and 6 with male) conducted not just with people who self‐injure (n = 9) but also those in a variety of relationships with people who self‐injure (n = 12), and those who have had both experiences (n = 6). In this way, we were better positioned to understand the interaction between self‐injury and the relationships that constitute its immediate social context.

It is important to emphasise that our purpose in this article is more theoretical than empirical. Our case studies are not intended to empirically establish our argument but rather help illustrate how a focus on relationships, interaction and communication has the potential to reframe our understanding and productively shape further research into the lived experience of self‐injury. We hope this theoretical article, using empirical examples for illustration, will demonstrate the value of the concepts that have informed our pilot study. And with this demonstration of concept established, we will provide a complete report on the empirical findings of our pilot study elsewhere.

Ultimately, we argue that we must reposition existing individualistic understandings of self‐injury within a broader social framework including a social communicative dimension. Challenging the prejudice of ‘attention‐seeking’ is important in itself as part of reducing the stigma experienced by people who self‐injure. But this challenge also helps us to explore how a broader framework could facilitate the development of alternative perspectives on self‐injury, and new avenues for sociological research.

## Violent communication

In her 1996 piece, Maggy Ross provides an interesting example of the paradox of the social in self‐injury:

I know why I self‐injure. I do it at times of extreme emotion: anger, self‐hatred, stress, grief and guilt. I do it to punish myself. When I feel I am losing control, I reach for a razor and prove to myself that I can, at least, have control over my body … The cuts are a visual expression of my distress. When I am lost for words, my cuts speak for me. They say – look – this is how much I'm hurting inside (1996: 13)

In the first part of this testimony, Ross provides a powerful but familiar description of troubling emotions and psychological motives, with an emphasis on the intrapsychic nature of self‐injury in stating that she reaches for a razor to ‘prove to *myself* that I … have control’ (our emphasis). But in the second part, her explanation becomes subtly less individual and intrapsychic in its language: her cuts are a ‘visual expression’ that ‘speak’ for her. What does it mean to think of this private act as an inherently communicative one? And if Ross’ self‐injury does indeed say ‘look – this is how much I'm hurting inside’ then who, on the outside, is this expression intended to reach? Whose gaze would recognise the ‘language of blood and pain’ and therefore understand and validate Ross’ expression of her inner state?

As already noted, such descriptions are not uncommon. Indeed, the social has always been something of an absent presence in self‐injury research. For example, with respect to *aetiology*, there is a well‐attested ‘role of pathological family relationships, parent–child discord and disrupted bonding in the risk of self‐harm’ (Gratz 2006: 239). Self‐injury has also been positively correlated with knowing other people who self‐injure (Hawton *et al*. 2006); a fact that has led to models of *social learning* (Hodgson 2004, Prinstein *et al*. 2009) and concerns over possible social ‘contagion’ within institutional settings (Crouch and Wright 2004) and through media exposure (Whitlock *et al*. 2009). Studies of social aetiology and social learning make an important contribution to understandings, but tend to leave the core intrapsychic model of self‐injury intact, and as such, the social paradox of self‐injury unresolved.

Building on these understandings, sociological work has contributed fuller, more sophisticated accounts of self‐injury in terms of sociocultural processes. Naturally these attempts have varied. At one end of the scale, for example, in Adler and Adler's work (2007, 2008, 2011), an emphasis on processes of *social construal* essentially maintains the intrapsychic model but argues that self‐injury can be framed and articulated in different ways as public understandings and representations change and develop. At the other end of the scale, for example, in Steggals’ work (2015), there is more emphasis on *social construction*, and the idea of self‐injury as a kind of symbolic practice or ‘idiom’ of disorder. Here, self‐injury is conceptualised as a culturally pre‐packaged pattern of meanings and actions that shapes and directs people's experiences and expressions of distress and social estrangement.

Other sociological work falling between these positions has focused on the narrative and phenomenological dimensions of experience (Chandler 2014, 2016, McShane 2012), or on patterns of individual practice (Brossard 2014). But again, this sociological work, while certainly developing our understanding of self‐injury in important ways, maintains a basic similarity to psy‐centric approaches and so fails to resolve the social paradox. Although taking different roads to get there, both psy and sociological perspectives converge on the image of a painfully isolated individual, alone behind their bedroom door, cutting themselves in secret and with little thought for *social communication* of any kind.

This conceptualisation is perhaps not surprising. Self‐injury *is* intensely personal; it is almost always conducted in private and frequently expresses feelings of social isolation (Smith *et al*. 1998, Solomon and Farrand 1996). But our suggestion here is not that this familiar conceptualisation is wrong, so much as it is partial and limiting. Ross’ passage, for example, invokes a powerfully expressive aspect, although not directly communicative and interactional. Indeed, self‐injury is often described through an expressive logic as a kind of symbolic externalisation; a *showing*, or making physical – making ‘real’ – a non‐physical and inner pain (Chandler 2016: 117, Steggals 2015: 89–96). People who self‐injure often question the validity of their inner experience, worrying that it does not describe real pain so much as reflect a narcissistic or histrionic defect of character. Expressing invisible inner pain as a visible bodily wound then, while acting as an emotional release, also provides a validation or authentication by drawing on the symbolic association of embodiment with the ‘real’ (Bendelow 2009). As Susan Kaysen, the author of *Girl, Interrupted*, puts it

I was trying to explain my situation to myself. My situation was that I was in pain and nobody knew it, even I had trouble knowing it … It [self‐injury] was the only way I could get through to myself … I was demonstrating, *externally and irrefutably*, an inward condition (2000: 153, our emphasis).

In this imperative, to *show* we encounter self‐injury as a kind of desperate bodily speech act, and uncover the core questions of the social paradox: does such an expressive action necessarily aim at social communication? If, by this recourse to the flesh, self‐injury is supposed to make a definitive statement, and so authenticate a particular kind of experience and identity, is it enough for it to be witnessed only by the person who self‐injures? Or is it something that ultimately (even if only ideally) seeks the validation of an‐other?

Certainly, Babiker and Arnold have argued that self‐injury is a form of self‐communication; that ‘[a]n individual may feel self‐injury to be a form of testimony; a way of being true to themselves and honouring their own experience [of past trauma]’ (1997: 79). And as Bartky notes in relation to feelings of shame: ‘I can become an object for myself; I can see myself as I might be seen by another, caught in a shameful act … the Other before whom I am shamed is only – myself’ (Bartky 1990: 85). But personal sentiments and the claims they make ‘are never quite real until they are exposed to the reaction of others and tested for truth and legitimacy in symbolic communication’ (Callero 2009: 51). A testimony is never fully a testimony until it has had its day in a public court. Self‐communication may be important then, but it may also be a partial position; a compromise between the desire to be ‘tested for truth and legitimacy’ on the one hand (self‐injury being recognised then, as a legitimate and *legitimating* testimony), and the fear of being found illegitimate and ‘attention‐seeking’ on the other.

In fact, there is ample evidence that the communicative dimension of self‐injury is, at least in part, something more than just self‐communication (Brown *et al*. 2002, Rodham *et al*. 2004, Turner *et al*. 2012). Nock and Prinstein (2004, 2005), for example, have argued that some self‐injury may represent a strategy for eliciting responses from others and for managing the social environment. Later work by Nock has argued that in many but not all cases, self‐injury may serve as a ‘high intensity social signal’ (2008: 159) where other possible types of communication or signalling (talking, yelling, crying) have not worked. This could happen where the signal is not strong, and the person who self‐injures has struggled with effective verbal communication, or in situations where the signal is poorly ‘detected’, perhaps because it is drowned out by the general communicative and affective noise of the social environment.

The possibility that self‐injury may serve interpersonal, interactional *and* intrapsychic functions, is evident in Hawton *et al*.'s (2006) schools study, which used a list of eight core motives to elicit participants’ reasons for ‘self‐harm’ (here implying both self‐injury *and* attempted suicide). While 72.8% of respondents identified intrapersonal motives, such as getting ‘relief from a terrible state of mind’, a significant minority identified more overtly interpersonal motives. These included ‘I wanted someone to know how desperate I was feeling’ (40.7%); ‘I wanted to find out whether someone really loved me’ (31.3%); ‘I wanted to get some attention’ (24%); ‘I wanted to frighten someone’ (21.1%) and ‘I wanted to get my own back on someone’ (14.3%) (2006: 55). Likewise, Spandler (1996) lists numerous core assertions made by her participants which express interpersonal and even interactional sentiments such as ‘I want someone to listen but I can't tell anyone’, ‘I want to talk about it but it's all about not being able to say anything and about there being no words for it’, ‘I want attention but I'm not worthy of it’, ‘I want people to notice and care but I hide it’ and ‘I want to approach people, ask for help, but that's a really hard thing to do and I can't guarantee it'll help’ (1996: 103–4).

This evidence supports a conceptualisation of self‐injury as involving a subtle and complex underlay of social and communicative, relational and interactional functions. Such a conceptualisation invites further exploration to enhance knowledge in this important area. Adding to sociological work on processes of social construal and social construction, this enhanced focus on social communication may be pivotal in dissolving the social paradox of self‐injury. However, though the observation of this underlay is well documented (Feldman 1988, Klonsky and Muehlenkamp 2007, Nock and Prinstein 2004, Prinstein *et al*. 2009, Walsh and Rosen 1988), robust examination of its implications has been lacking. This may be because such questions are more sociological than psychological, but it may also reflect an (over)sensitivity to the potential for such conceptualisation to stray into accusations of ‘attention‐seeking’ (Chandler 2016).

## Attention‐seeking, recognition and visibility

The phrase ‘attention‐seeking’ has negative and histrionic connotations, implying an unwarranted method for extracting kindness, sympathy and the benefits of the ‘sick role’ (Parsons 1951). Indeed, some of these connotations connect self‐injury with the historical idiom and discourse of hysteria in a complex and contested genealogy of personal distress and social discrimination (Steggals 2015: 33–5). Like hysteria, self‐injury is culturally coded as being *essentially feminine* regardless of the biological sex or gender identity of the person self‐injuring (ibid.). And just as ‘attention seeking’ has been applied as an unrestricted generalisation enabling dismissive views on self‐injury (Crouch and Wright 2004), the same can be said for young women in general, who constitute both the biggest demographic group of people who self‐injure, as well as the popular stereotype of ‘the self‐harmer’. But as Louise Pembroke counters with considerable power:

What is attention‐seeking? I know that I do not seek the degradation I have received in Accident and Emergency, neither do I want to be treated with sympathy nor pity … If I wanted ‘attention’ in an exhibitionist way, it would be much easier and pain‐free to walk into the middle of the street and remove my clothes. I would not need to cut up my body’ (1996: 44‐45)

Most user groups, such as the *National Self‐harm Network* and *Self‐injury Support*, and many writers (McAllister 2003) regard the idea of attention‐seeking as a pernicious myth. As Pembroke suggests, the implicit moral assessment of the phrase ‘attention‐seeking’ invokes connotations of psychological weakness and dependency, mixed with a fraudulent and selfish social manipulation. The resulting sensitisation to any suggestion that self‐injury is anything but wholly individual, secret and private is understandable in this context. Indeed, many see the privacy of the practice as the guarantor of its authenticity (Chandler 2016), with non‐private elements leading to categorisation as inauthentic and attention‐seeking (Scourfield *et al*. 2011). If self‐injury is in part then, as we have suggested, an attempt to communicate and hence *authenticate* a particular kind of inner experience, it is peculiarly frustrated by the fact that any such communication can all‐too‐easily be read as evidence that no ‘real’, authentic – and hence *authenticating* – self‐injury has occurred.

Still, as Chandler has noted ‘[s]cratch the surface, and the idea of ‘private’ self‐injury becomes contested: private to who, secret from who?’ (2016: 197–98). Reversing the standard framing, she suggests that ‘the continued valorisation of privacy and secrecy maintains a cultural account where self‐injury is viewed as private, secret, *and therefore* visible self‐injury is subject to negative readings: “manipulation”, “attention‐seeking” – “inauthentic”’ (2016: 198, her emphasis). So, while self‐injury – or at least a claim to it – as a kind of social manipulation is conceivable (and the status it now carries in the public imagination does make it available for multiple aesthetic or identity projects) it nonetheless seems likely that if someone is willing to mutilate themselves then they are probably motivated by a significant felt need, regardless of how secret they keep their injury.

Pembroke also reframes the issue, arguing: ‘if “attention” means being listened to and taken seriously, then along with the rest of the human race I'm attention‐seeking’ (1996: 44–5). She continues ‘consideration should be given to what attention [a person who self‐injures] … needs’. For some people self‐harm is a form of communication to voice things that cannot be said (1996: 45). Testimony from Spandler's study supports this interpretation:

I just wanted someone to listen … [but] no‐one really knew about it because I hid it … All I wanted was someone to listen, to be taken away from the situation, someone to notice without me having to tell them. You need attention – there's something drastically wrong but you can't actually say to anyone ‘look I need your help’ so you tell in another way (1996: 105‐6)

Far from implying histrionics then, ‘attention’ could be something that, as one participant from Steggals’ study describes it, exists between ‘the desire for self‐effacement and the desire, not to be looked at exactly, but just to be *recognised*’ (2015: 160, our emphasis). Recognition has more usually been considered in terms of claims to be considered a full and worthwhile member of a polity (Fraser and Honneth 2003; also Lawler 2005). But while one set of claims to be taken seriously may be made on the basis of citizenship, and another set of claims may be made, as here, on the experience of authentic and authenticating distress, there is a sense in which both cases imply a deeper demand: that the subject be recognised, as Arthur Frank puts it in his discussion on recognition, ‘as fully human’ (1991: 87). As one person put it to Hewitt: ‘[c]utting seems to be a great self‐destructive attempt to become human. To gain recognition, to prove to someone that I matter, and that I bleed too’ (1997: 57).

Self‐injury then, rather than being attention‐seeking in the pejorative sense, is better understood as containing a powerful and desperate self‐communicative and social communicative imperative; and it may in fact be attention, *as recognition*, which is desired and demanded (Crouch and Wright 2004). The complication is that this desire is likely to be frustrated in contexts where obviously communicated self‐injury is misinterpreted as evidence of inauthenticity. Under such conditions self‐injury can become, as Steggals notes

like a letter that has been written but not sent, set aside in a safe place with the possibility that it may be sent in the future and with the hope that by then the recipient will be able to understand it, to recognise the truths that it contains … The writing of this letter, of inscribing ‘please help me’ onto the body, would seem in and of itself to often be enough, at least for the immediate psychological needs of the person (2015: 160)

This may be true, but it is also true that the letter, even if it is kept hidden for a while, does seem to have a habit of being at least shared, and perhaps even delivered. Most self‐injury does not remain strictly secret (Muehlenkamp *et al*. 2012) but becomes subject to a complex and ambiguous play of revealing and concealing, or what Chandler calls ‘visibility’ (Chandler 2016: 109–44). The underlying logic of this process is that an act of showing enables communication, but works *only* because it is not framed as a deliberate act of communication or ‘attention‐seeking’ (i.e. it shows, but it is not *seen to show*).

Most forms of self‐injury leave marks on the skin, which already endangers secrecy through their potential visibility. In addition, self‐injury is often enacted ambiguously across the borders of the inner/outer divide, sensitising the public/private boundary. Brossard (2014) for example, talks about ‘virtual opportunities to “seek help”’ (2014: 568) where those about to self‐injure stage imaginary social scenes in which their intentions are disclosed in the hope of eliciting understanding and even intervention. He reports that some of his participants might go to the supermarket to buy razorblades, hoping that the cashier would realise what they were doing and intervene (ibid). We could add other examples: using unconvincing explanations for visible scars (an infamous example being: ‘the cat did it’); leaving bloody clothes in a shared laundry basket; leaving out blood‐stained tissues and towels; inadequately hiding self‐injury kits; seeking medical attention for physically superficial wounds; injuring oneself in public restrooms; wearing clothes that reveal scars; or, conversely, hiding scars in highly conspicuous ways such as under heavy, long‐sleeved clothing on hot summer days. Chandler talks about how she maintained a ‘self‐narrative that I had ‘always’ kept my self‐injury secret’ nonetheless:

[a]t one point, in my mid‐teens, I had cut my face and hand – injuries that were inevitably seen by others. Further, throughout the time I injured myself, the wounds would be ‘revealed’ on occasion, sleeves would ride up my arm, people noticed, stories of explanation had to be provided, cats were blamed (2016: 134)

She concludes that although ‘I had been clear my injuries were never about ‘attention‐seeking’ – they nonetheless did attract ‘attention’ and were not always ‘hidden’’ (2016: 134). In addition to such examples, it should also be noted that many people who self‐injure participate in digitally mediated communities in which they may publicly discuss and even display their self‐injury (Adler and Adler 2008, Whitlock *et al*. 2009). The presence of self‐injury–related content on social media platforms is of course a controversial and contested issue, as highlighted by the recent Instagram ban on self‐injury images (Chandler 2019), but it is also a useful reminder that this personal practice can include a definite public dimension.

These twin issues of recognition and visibility then, while present as observations in research findings, are largely absent from explanatory models of self‐injury. Together these concepts help demonstrate that self‐injury is a complex area of social practice that works across and between the borders of the inner/outer and private/public distinction, and even the intentional/unintentional. The social politics of recognition, and the play of visibility, suggest that ambiguity and ambivalence play a critical role in how self‐injury is conceptualised and undertaken. Revealing and concealing do not form a discrete binary choice (Chandler 2016), but rather represent two poles in personal dilemmas where agency and action take on a confused and confusing indeterminacy. We argue that self‐injury undermines the strict demarcation of the psychological from the sociological, and indeed undermined any straightforward inside/outside dichotomy. Self‐injury is a personal act, yet simultaneously a part of a person's relational life and interwoven with the interaction order: that domain of virtual or actual co‐presence, structured by social rules that are largely unthinkingly observed (Goffman 1983). Self‐injury is therefore a rich resource for sociological investigation, if we approach it as something occurring within, and having effects across, networks of relationships.

To illustrate this key argument and the themes that support it, we utilise here two case studies. These case studies help to demonstrate the need for a sociology of the social communicative dimension of self‐injury, and to contextualise our theoretical discussion in the context of lived experience. Here, we shall briefly examine the case of Debra, a 23‐year‐old woman who reports self‐injuring between the ages of 12 and 15, and then again from 18 to 22; and that of Rachel, a 47‐year‐old school nurse, and the mother of two daughters, the younger one of whom, Mia, has a history of self‐injury that began when she was 13 or 14 years old (these case studies have been anonymised). These two particular cases have been selected because, while Debra and Rachel are unacquainted, their cases mirror and reflect one another from both sides of self‐injury's social equation: Debra as a young teenager beginning to self‐injure, and Rachel as the concerned mother of a young teenager who is self‐injuring. Of course, real cases are always multi‐dimensional, the product of a complex interaction of factors, with many possible aspects that could be explored further. However, our interest here is strictly illustrative: to show how the argument we have developed here, through a critical engagement with existing research, has significant potential to inform a more nuanced analysis of lived experiences.

## Case Study 1: Debra

For Debra, her self‐injury is ‘definitely tied into’ her relationships. She describes three close relationships that produced emotions that were both strong and frightening to her. They were emotions that she struggled to ‘process’, threatening to overwhelm her sense of self‐control and even her sense of individual selfhood. The emotional product of these relationships was often anger, which she controlled through acts of self‐injury, although other factors were also important, including ‘the big questions’ about what she will do with her life, and the sense of being ‘lost’ that comes with not knowing the answer. Her self‐injury began at school and was especially connected with her ‘obsessive’ and troubled relationship with her best friend who also self‐injured. A period of little or no self‐injury between the ages of 15 and 18 was initiated by her breaking off this relationship. She began self‐injuring again when she went to university, following a short intimate relationship which she reports ‘did not go well at all and kind of damaged my self‐esteem’, and continued through a longer intimate relationship that had ended a year before her interview.

When Debra first began to self‐injure, she tellingly tried to keep it a secret from everyone *but* her best friend. ‘I wanted her to see’ she explains

But I was hoping as well that it would bring us closer together or, or something … or just to get attention from her I guess or, um, yeah, some kind of affection. Yeah, it was strange that it was sort of a way that we used, we used it as a way to get affection from people; so, we wanted people to feel sorry for us … [just] so they know that you're suffering I guess … It's a wordless way of showing that you're not okay.

Debra's wonderfully neat description of self‐injury as ‘a wordless way of *showing*’, highlights the social communicative function that lies behind it. In response to the question ‘what's wrong with words?’ Debra describes what Kilby (2001) has called the ‘failed promise of language’, the sense of being unable to articulate, and as such understand, feelings in the terms provided by conventional language. She traces this to her childhood, and explains that, following the death of her mother's partner: ‘I kind of went through this trying‐not‐to‐show‐any‐emotions phase’.

Steggals Yeah, was that sort of being strong for your mum or … ?

Debra Yes, I think so, because a lot of people told me in that period that that's what I should be. Um, and that I should look after her and stuff and I kind of, yeah, I guess I took that on at nine‐years‐old which then led to not being able to deal with emotions later in life … on our whole, er, mum's side of the family it's very um, not talking about emotions. That's always been quite a big thing and even now, like, we don't talk about things that involve emotions and I guess that fed in, in a way, to my not being able to process emotions, and that's what potentially led me to er, use, yeah, harming as a way of feeling something.

Arthur Frank (1991), in describing the ‘communicative body’, explains that when ordinary language fails in this way, it is typically the body that ‘breaks out of [the] codes’ that have silenced the subject, seeking instead to ‘find self‐expression in a code of its own invention’ (1991: 85). Or, as Hewitt puts it: ‘gesture replaces language. What cannot be said in words becomes the language of blood and pain’ (1997: 58): a wordless way of showing. Certainly, what is being shown here is a testimony of suffering, of what Debra feels is her authentic inner truth. But interestingly, in the context of talking about one of her later intimate relationship, the purpose of this showing appears to go beyond recognition alone, and implies recognition in service to a social and emotional bond.

Steggals But you were saying about showing somebody the depth or strength of your feeling as well; is part of it wanting him to see, in a way, what he had done to you or how much you felt?

Debra Yeah, and, yeah; how I was hurting. Um, I guess, I don't know if to a rational person that, like now I know that that's not something that's ever going to work to make somebody love you, but I guess as an irrational person, um, you kind of do anything don't you?

Both this later intimate relationship, and her earlier intense relationship with her best friend then (as well as Debra's understanding of her best friend's own self‐injury), were characterised by experiences of self‐injury focused on eliciting ‘some kind of affection’. This raises an interesting question: is the desire for recognition not only about a sense of self‐validation but also the particular person you want validation from? Could this desire, and the sharing of the secret of self‐injury, serve to (re)negotiate and strengthen a social bond? To explore this further, we turn to our second case study.

## Case Study 2: Rachel and Mia

Rachel participated in an interview as the mother of Mia, who had a history of self‐injury. Mia's self‐injury was therefore described to Steggals by Rachel, who characterised it as part of Mia's broader pattern of unhappiness including depression, two suicide attempts, and a period of food‐refusal. Rachel was uncertain about what had caused this unhappiness, although she said that Mia has always maintained that it was the product of peer bullying. Rachel's account certainly echoes the ‘failed promise of language’ as her earlier relationship with Mia disintegrated, and both communication and contact between them began to break down. Indeed, the most striking element of Rachel's story is how pronounced these issues of communication are, as alternative forms of communication evolved to fill the awkward social space that opened‐up between them. Mia's self‐injury is the obvious first example, the recourse of Frank's communicative body, mediated by the ambivalent and ambiguous displays of visuality noted earlier. As Rachel tells the story of how she discovered her daughter's self‐injury:

She was lying in the garden with um, she got, she'd been upstairs, got changed and she'd got a little strappy top on, and she'd got her arms behind her head, lying in the sun. And I walked up to her and I thought ‘oh my gosh she's got, you know, she's got scarring down her arms’ … She knew I'd clocked it, she'd know, but she'd obviously, she'd put a strappy top on, so she must have known I was going to see that day … Um, so you know, whether that, that element was, you know, she was ready to, to share that information with me I guess.

But while Mia's self‐injury may have been known, it was not public: Rachel immediately acted to keep the fact from her own mother, as well as Mia's sister and father. The ideal poles of *secret and shared* then, do not map perfectly onto the equally ideal poles of *private and public*, and a shared secret is still a secret nonetheless. But even within the context of this shared secret, communication between Mia and Rachel was not yet restored. Mia would not speak directly about her issues with Rachel, although she did begin to send messages through her CAMHS worker. And for her part, Rachel found that she could not speak about the self‐injury.

I couldn't really say the words when I first found out, I couldn't use the word ‘self‐harm’, I couldn't, or ‘hurt yourself’, or ‘cut yourself’; I couldn't use those words, they couldn't come out of my mouth, they were too painful

The failure of language spread then, with the secret itself, from daughter to mother. But, in time a new form of communication developed. Rachel, on the pretext of cleaning Mia's room, would search for hidden razor blades and would let Mia know what she had found. While these items were hidden, the fact that Mia knew her mother was going into her room, taken together with the fact that these items continued to be left there, raises some of the same issues around ambiguous communication and visuality that Mia's original display of her self‐injury also raised. Indeed, Mia told her CAMHS worker to tell Rachel where to find some old suicide notes that she had written. In this way, Mia's bedroom became a kind of alternative bulletin board in which things left, and things discovered allowed for some controlled and largely wordless way of showing; an alternative mode of communication.

In time Rachel and Mia developed a more sophisticated ‘wordless way’ of communication that Rachel refers to as ‘the code’:

So, she would send me, we had codes, so if she was going to cut at night she would send a code on my, by text, to say she was feeling like she needed to cut. Um, and we had an agreement at that point that, if she sent me that text, I'd go upstairs to her and, you know, we'd try some strategies.

[W]e had a little code system for all of it. And we devised it together. So, it was ‘sad face’ if she was thinking about self‐harm, or, you know, it was a kiss, an ‘X’ if it was something else. And we, it was a Morse, it was almost like a Morse code.

This code, delivered by text or by leaving notes on the kitchen table, provided different symbols, resulting in different prearranged responses and strategies. This brilliantly provided them with a wordless form of communication and coordination that circumvented the issues that had come to weigh down ordinary language. As Rachel explains:

I couldn't say the words, I couldn't say the words and I couldn't talk about it … these words are really hard to say out loud, and if I can't say them, you may not be able to say them either.

Over time the code allowed Rachel to feel a new confidence in her relationship with her daughter, and even helped Mia to stop self‐injuring. As Rachel explains this, she says that people who self‐injure ‘can't express [their feelings] in words and [their self‐injury is] a strategy they're using, so actually, we found a different strategy’.

Returning to the question we were left with at the end of Debra's story, Rachel believes this renegotiation of the social and emotional bond between her and Mia certainly did strengthen their relationship:

it was almost really special. And I'd, I'd say that um, also, I actually felt I grew a lot closer to her … I actually feel closer. There's an ultimate bond there, now, or another bond, on another level … I do feel that I, actually, again, the silver lining is that I did form a different relationship, and a much more honest relationship.

## Conclusion

Whilst illustrative case studies do not allow the same level of theoretical generalisation as an inductive analysis of a broad data set, they do facilitate a more in‐depth demonstration of the conceptual argument we have presented; and thereby provide insight into how the analysis of empirical data could be developed by reframing self‐injury as – at least in part – an issue of social communication. What Derbra and Rachel's testimony illustrates – through the failure of ordinary language, its replacement by the alternative semiotics of the communicative body and the renegotiation of language (and relationships) through other forms of expression – is that self‐injury is intimately, although not exclusively, connected with issues of both self‐ and social communication.

The paradox of the social in self‐injury then, is a tension that is likely produced more by how we frame the practice than by anything inherently confused and confusing about self‐injury. If our ability to resolve the paradox is limited by the overly intrapsychic models that dominate our thinking, then it is this way of modelling both self‐injury and subjectivity that we must overcome. The sociological literature has already begun this work, but the presence of social communication in self‐injury suggests that we need to go much further. We need to understand self‐injury as something that is *not only social in its formation but also in its ongoing daily practice*. But we also need to disrupt the traditional Western model of subjectivity that informs the exclusively intrapsychic paradigm (Chandler 2016, Steggals 2015). The monadic, self‐sufficient individual, or *homo clausus* (Elias, 2000 [1939]), and the whole binary structure of inner/outer, individual/social and private/public that goes with it (Callero 2009, Derrida 1982) must be reframed as *patterns of enacted and embodied values* rather than as ontological givens.

As we have seen, one possible model to shape this further work would be Frank's concept of the ‘communicative body’ (1991), in which the body, contra *homo clausus*, is essentially ‘dyadic’, socially ‘contingent’ and ‘other‐related’. Echoing Nock's observations about ‘high intensity social signals’, Frank argues that it is when narratives of silenced – which is to say, *unrecognised* – selfhood, of vulnerability and suffering, ‘*are spoken from … the body* that they can be shared most readily’ (1991: 89, our emphasis). Perhaps this is precisely because the language of the body is, as we have seen, located ambiguously between intention and accident; between conscious (and hence deliberate) action, and unconscious (non‐deliberate) symptom. There is anxiety associated with the dilemma of disclosing oneself in the hope of recognition on the one hand, or else concealing oneself to avoid a failure of recognition – and the rejection of self that this implies – on the other. This anxiety may be partially managed by using a bodily strategy of expression (self‐injury) that is indeed communicative, but that falls short of being a fully deliberate, intentional communication. Only further sociological research into the social communicative dimension of self‐injury will improve our understanding. And such sociological work also carries clear implications for practice, especially if it can be carried out in partnership with clinicians. Indeed, a deeper understanding of the communicative dimension of self‐injury will help inform clinical approaches to care, such as integrating personal coping strategies with those based on people's available social support network. Such research is clearly needed. Whenever the body speaks it is important, and however the body is speaking in self‐injury, it seems clear what the message is: I'm in pain, I matter, I deserve recognition.
